# Recovery of serum testosterone levels is an accurate predictor of survival from COVID-19 in male patients

**DOI:** 10.1186/s12916-022-02345-w

**Published:** 2022-03-29

**Authors:** Emily Toscano-Guerra, Mónica Martínez-Gallo, Iria Arrese-Muñoz, Anna Giné, Noelia Díaz-Troyano, Pablo Gabriel-Medina, Mar Riveiro-Barciela, Moisés Labrador-Horrillo, Fernando Martinez-Valle, Adrián Sánchez Montalvá, Manuel Hernández-González, Ricardo Pujol Borrell, Francisco Rodríguez-Frias, Roser Ferrer, Timothy M. Thomson, Rosanna Paciucci

**Affiliations:** 1 Biochemistry Service, Vall d’Hebron Hospital, Autonomous University of Barcelona (UAB), Barcelona, Spain; 2 Cell Signaling and Cancer Progression Laboratory, Vall d’Hebron Institute of Research (VHIR), Barcelona, Spain; 3 Universidad Peruana Cayetano Heredia, Lima, Perú; 4 Immunology Division, Vall d’Hebron Hospital, Barcelona, Spain; 5 Translational Immunology Research Group, Vall d’Hebron Research Institute (VHIR), Barcelona, Spain; 6 Department of Cell Biology, Physiology and Immunology, Autonomous University of Barcelona (UAB), Barcelona, Spain; 7 Internal Medicine Service, Vall d’Hebron Hospital, Barcelona, Spain; 8 Infectious Diseases Department, International Health and Tuberculosis Unit National Referral Centre for Tropical Diseases, Vall d’Hebron University Hospital, Vall d’Hebron Institute of Research (VHIR), Barcelona, Spain; 9 Barcelona Institute for Molecular Biology, National Science Council (IBMB-CSIC), Barcelona, Spain; 10 Networked Center for Hepatic and Digestive Diseases (CIBER-EHD), Instituto Nacional de la Salud Carlos III, Madrid, Spain; 11 Plataforma Temática Interdisciplinar Salud Global (PTI-Global Health) CSIC, Madrid, Spain

**Keywords:** COVID-19, Survival, Longitudinal, Testosterone, Immune phenotype

## Abstract

**Background:**

SARS-CoV-2 infection portends a broad range of outcomes, from a majority of asymptomatic cases to a lethal disease. Robust correlates of severe COVID-19 include old age, male sex, poverty, and co-morbidities such as obesity, diabetes, and cardiovascular disease. A precise knowledge of the molecular and biological mechanisms that may explain the association of severe disease with male sex is still lacking. Here, we analyzed the relationship of serum testosterone levels and the immune cell skewing with disease severity in male COVID-19 patients.

**Methods:**

Biochemical and hematological parameters of admission samples in 497 hospitalized male and female COVID-19 patients, analyzed for associations with outcome and sex. Longitudinal (in-hospital course) analyses of a subcohort of 114 male patients were analyzed for associations with outcome. Longitudinal analyses of immune populations by flow cytometry in 24 male patients were studied for associations with outcome.

**Results:**

We have found quantitative differences in biochemical predictors of disease outcome in male vs. female patients. Longitudinal analyses in a subcohort of male COVID-19 patients identified serum testosterone trajectories as the strongest predictor of survival (AUC of ROC = 92.8%, *p* < 0.0001) in these patients among all biochemical parameters studied, including single-point admission serum testosterone values. In lethal cases, longitudinal determinations of serum luteinizing hormone (LH) and androstenedione levels did not follow physiological feedback patterns. Failure to reinstate physiological testosterone levels was associated with evidence of impaired T helper differentiation and augmented circulating classical monocytes.

**Conclusions:**

Recovery or failure to reinstate testosterone levels is strongly associated with survival or death, respectively, from COVID-19 in male patients. Our data suggest an early inhibition of the central LH-androgen biosynthesis axis in a majority of patients, followed by full recovery in survivors or a peripheral failure in lethal cases. These observations are suggestive of a significant role of testosterone status in the immune responses to COVID-19 and warrant future experimental explorations of mechanistic relationships between testosterone status and SARS-CoV-2 infection outcomes, with potential prophylactic or therapeutic implications.

**Supplementary Information:**

The online version contains supplementary material available at 10.1186/s12916-022-02345-w.

## Background

The COVID-19 pandemic, caused by SARS-CoV-2, is characterized by a diversity of clinical manifestations, including exacerbated inflammatory states accompanied with tissue and organ destruction beyond direct viral cytopathic effects. From the outset of the pandemic, it became clear that, while men and women present a similar prevalence of infection [1, 2], a higher risk of severe disease and death is significantly associated with male sex [2, 3], similar to the other pathogenic coronaviruses, SARS-CoV and MERS-CoV, and other viral respiratory infections [4, 5]. Multiple factors have been considered to explain the sex disparity observed in the development of severe COVID-19, including differential biological and pathophysiological impacts of age and comorbidities such as cardiovascular disease, high blood pressure, chronic obstructive pulmonary disease (COPD), diabetes, obesity, or active cancer [6]. Gender differences in disease severity are also observed in racial and ethnic minority groups, disproportionately affected by COVID-19 [6].

The underlying mechanisms that may account for the differences between men and women in the development of severe COVID-19 are not completely understood. As the main cellular receptor for SARS-CoV-2, ACE2, and the major viral fusogenic membrane-associated protease, TMPRSS2, are under transcriptional regulation by androgens [7], it had been predicted that men would present a higher propensity of infection by SARS-CoV-2 and to develop more severe disease than women [7, 8]. However, men and women show comparable risks of infection [1, 2] and observational studies of male COVID-19 patients under androgen-deprivation therapy have yielded contrasting results with regard to risk of developing severe COVID-19 (ref. [9, 10]). Contrariwise, there is growing evidence that severe COVID-19 in male patients is accompanied with diminished levels of circulating testosterone [11–13], suggesting a critical role for androgens [11, 14] and androgen receptor, AR [15], in preventing the innate and/or adaptive immune dysfunctions that lead to the development of severe forms of the disease [16–18]. A role on coagulation for testosterone has also been suggested as a factor to consider in COVID-19 pathogenesis [19, 20].

The sexual dimorphism of immune responses to pathogens has been long recognised [21], pointing to women having stronger antiviral mechanisms, stronger T regulatory cells, higher numbers of group 2 innate lymphoid cells (ILC2), and superior immune-mediated tissue repair capacities as compared to men [22, 23]. In addition, sex hormones may differentially impact the frequency and severity of many autoimmune and inflammatory diseases, generally more prevalent in women than men [24, 25]. It should be noted that sex hormones may exert apparently contrasting effects. For example, an immunosuppressive role for testosterone was observed in response to influenza vaccination [26], while testosterone supplementation following influenza infection in aged male mice, which caused decreased serum testosterone levels, reduced mortality [27].

In order to better understand the relationship between testosterone status and disease severity, we have analyzed serum biomarkers, including testosterone, and immune cell subpopulations in COVID-19 patients in association with disease outcome. We have found that the trajectories of serum testosterone levels are highly accurate predictors of survival in male COVID-19 patients. Furthermore, we establish that male COVID-19 patients with a fatal outcome display a late coordinated depletion of circulating subsets of differentiated CD4+ T lymphocytes and monocytes, mirrored by a relative enrichment of undifferentiated CD4+ T cells and monocytes.

## Methods

### Study design

With the aim of exploring factors that may underlie worse progression of COVID-19 in men, we have undertaken a retrospective study with 497 (249 males and 248 females) patients admitted to the Vall d’Hebron Hospital (HVH) between May1st and June 30th 2020, with RT-PCR-confirmed diagnosis of SARS-CoV-2 infection. Patients were first studied for serum biochemical and hematological variables in samples collected at or near admission (first time point or Sample 1), for baseline assessment. Subsequently, a subcohort of 114 male patients was studied for the progression of the disease by analyzing the same variables longitudinally, and samples from this subcohort were further analyzed for serum luteinizing hormone (LH) and androstenedione levels, as well as an extensive phenotyping of circulating immune cells.

### Patient selection

Patients included in the study were consecutive patients admitted to the HVH, with the following exclusion criteria: previously hospitalized, recently transplanted, immunosuppressed, and hormonally depleted. One hundred fourteen male patient subcohorts studied for longitudinal analyses were chosen on the basis of serum sample availability. Within this subcohort, a group of 24 patients, for whom matched serum and peripheral blood mononuclear cells were available, was studied for immune phenotyping.

### Patient classification

Patients were classified as mild, moderate, severe-survivor, and severe-deceased as per a 4-point scale adapted from the 6-point ordinary scale used by Grein et al. [28] as follows (Table ST3): Mild: symptomatic patients with PCR-diagnosed SARS-CoV-2 infection that were in emergency unit or required hospitalization for less than 2 weeks; Moderate: hospitalized patients requiring low-flow oxygen (mask or nasal prongs) or high-flow oxygen, not requiring ICU admission; Severe-survivor: patients admitted to the ICU requiring non-invasive or invasive mechanical ventilation, then discharged; and Severe-deceased: ICU patients with a fatal outcome.

### Data and sample collection

The final dataset was a compilation of data from the Vall d’Hebron Laboratory database and the Vall d’Hebron prospective COVID-19 cohort database, which was collected prospectively from medical doctors during the first and second wave of the pandemic using a case report form (CRF) designed by the Infection Disease Department in RedCAP web-based platform. Comorbidities considered were chronic lung disease, cardiovascular disease, diabetes, chronic kidney disease, liver disease, HIV infection with good adherence, obesity (BMI ≥ 30), and cancer. For single-point analyses, data were obtained on (or near) hospital admission date. For longitudinal analyses, data were obtained throughout hospitalization with serial time-point sample collection every 7 days in average. For patients in the severe outcome groups, up to five time-point samples were analyzed. Patients in the combined mild-moderate outcome groups were discharged in average, after 2 weeks of admission, and a maximum of three time-point samples were procured from them. For immune phenotyping analyses, at least two independent samples (Sample 1 and Sample 2) were collected, separated by 5 to 20 days.

### Serological determinations

Serum biochemical variables were measured by automated analyzers at the Biochemistry Service Core Laboratory Facility at HVH. All determinations were compared to internal controls used for reference ranges at the Core Facility. Serum hormone levels were determined by chemiluminiscent immunoassays (CLIA) on an AtellicaTM IM Analyzer (Siemens Inc., NY), using testosterone TSTII (Siemens ref. 10995707) and luteinizing hormone (LH) (Siemens ref. 10995634) kits. Androstenedione was measured on a LIASON XL Analyzer (DiaSorin, Saluggia, Italy), using the LiasonR androstenedione (ref. 318870) assay. As references for healthy men, we used median total serum testosterone levels of 409.72 ng/dL (90% CI 197.44–669.58) for < 50-year-old individuals and 377.46 ng/dL (90% CI 187.72–684.19) for ≥ 50-year-old individuals (FDA-approved protocol, https://www.accessdata.fda.gov/cdrh_docs/pdf19/K191533.pdf). For luteinizing hormone (LH), the reference median values were 2.8 mIU/mL (90% CI 1.5–9.3) for < 70-year-old individuals and 8.0 mIU/mL (90% CI 3.1–34.6) for ≥ 70-year-old individuals. For androstenedione, the reference median value was 1.80 ng/mL (90% CI 0.5–3.5). Bioavailable-free testosterone was calculated according to Vermeulen et al. [29].

### Immuno-phenotyping

Blood samples were collected in Vacutainer tubes containing ethylene-diamine-tetra-acetic acid (EDTA) as anticoagulant (BD-Plymouth, PL6 7BP, UK) and processed within 4 h after collection. Absolute counts and relative numbers of peripheral blood lymphocytes were determined for all study participants using tetra CHROME Tube 1 (CD45-FITC/CD4-PE/CD8-ECD/CD3-PC5) and tetra CHROME Tube 2 (CD45-FITC/CD56-PE/CD19-ECD/CD3-PC5) panels (Beckman Coulter). Samples were fixed in 1X lysing solution (BC) and acquired on a BC Navios EX instrument.

For multicolor staining and analysis, extended lymphocyte subpopulations were assessed with 5 different flow cytometry panels designed according to the HIPC protocol [30]. Two additional panels were added to analyze basic lymphocyte populations and RTE. Compensation controls were used in each panel to avoid overlapping of the different fluorochromes. Gating strategies were as described [31], and the antibody panels were summarized as follows and in Table ST4:

- Panel 1: General immune phenotype for T, B, and natural killer (NK) lymphocyte subpopulations, gating by CD45 versus SSC.
- Panel 2: Gating strategy for differentiated CD4^+^ and CD8^+^ T cell subsets, based on CD45RA and CCR7 expression defining: CD45RA^+^/CCR7^+^ (naïve), CD45RA^−^/CCR7^+^ (central memory [TCM]), CD45RA^−^/CCR7^−^ (effector memory [TEM]), and CD45RA^+^/CCR7^−^ (terminal effector memory [TEMRA]). CD4^+^ T helper (Th) populations (Th1, Th2, Th17, Th1–17), based on CCR6 and CXCR3 expression, were analyzed by gating on CD45RA− TCM and TEM cells.
- Panel 3: T regulatory (Treg) cell populations: CD3^+^CD4^+^CD25^+^, CD127^−^, CCR4^+^, and CD45RO^+^.
- Panel 4: B cell populations (naïve, pre-switched, switched memory, and exhausted) depending on expression of IgD and CD27. The differing pattern of CD24^+^ and CD38^+^ expression identified transitional cells and plasmablasts. CD27 and CD21 enabled study of the CD21low population.
- Panel 5: Dendritic cells (DC), natural killer (NK) cells, and monocyte populations were analyzed in the CD3^−^CD19^−^gate. NK subpopulations (NK^dim^ and NK^bright^) were studied using CD56 and CD16 expression. CD16 and CD14 were used to identify classical monocytes (CD14^+^CD16^−^) and non-classical monocytes (CD16^+^CD14^−^). DCs were studied selecting for populations negative for the following markers: CD3, CD14, CD16, CD19, CD20, and CD56. High expression of HLA-DR and CD11c and CD123 was used to identify plasmacytoid DCs (HLA-DR^+^CD123^+^) and myeloid DCs (HLA-DR^+^CD11c^+^).
- Panel 6: Recent thymic emigrant cells (RTEs) were studied using CD3, CD4, CD27, CD31, CD45RA, and CD62L expression.

Data were acquired on a NAVIOS EX (BC) flow cytometer. At least 100,000 events were acquired for each sample. Flow cytometry data were analyzed with Kaluza Software. Absolute values were calculated from the absolute number of leucocytes and lymphocytes as determined on a hematological analyzer (XN-2000; Sysmex, Japan).

### Statistical analysis

Continuous variables were expressed as mean ± SD or median and interquartile range (IQR). Simple and multiple comparisons were performed using parametric (two-sided Student’s *t* test or ANOVA) and non-parametric (Mann-Whitney *U* test or Kruskal–Wallis) statistical tests with Dunn’s and Tukey’s post hoc tests. Categorical variables were presented as numbers and percentages and compared using the 2-sided Fisher’s exact test as appropriate. Correlation between variables were assessed using simple linear regression. ROC curves were calculated with the univariate logistic regression model implemented in GraphPad and the EasyROC web tool (http://www.biosoft.hacettepe.edu.tr/easyROC/). The groups classified by the model were used in 2 × 2 contingency analysis to calculate odds ratios (OR) and significance determined by Fisher’s exact test. For longitudinal analyses, trajectories were plotted for each patient and average values for each parameter calculated for each time-point, followed by linear regression. The resulting linear regression slope values were used in univariate logistic regression analysis to assess the outcome predictive power of the trajectories. *P* values ≤ 0.05 were considered significant. For principal component analysis (PCA), the variables analyzed included all the biochemical parameters and immune subpopulations, including numerical assignments for outcome (1, mild–moderate; 2, severe; 3, deceased) and selecting for ≥ 3 components, of which the 2 summarizing the highest variance were used for the 2-dimensional representations. PCA, multivariate correlation analysis, and other calculations, as well as graphic representations, were performed with GraphPad Prism 9.0.2.

### Ethical considerations

The present study was performed with surplus serum samples from routinely tested hospitalized COVID-19 patients, following protocols reviewed and approved by the HVH Institutional Review Board (Medical Research Ethics Committee, protocol number PR(AG)329-2020). Immunophenotyping studies of the peripheral blood cells underwent a separate review and approval process (protocol number PR(AG)242/2020). For the final dataset from the Vall d’Hebron prospective COVID-19 cohort database, clearance from the Institutional Review Board was obtained. To minimize risks of infection to the health staff, a written informed consent was waved, although all patients received proper study information and gave oral consent.

## Results

### Biochemical and hematological predictors of outcome in male and female COVID-19 patients

Biochemical and hematological parameters were analyzed at admission for 497 male and female COVID-19 patients. Demographics, background information, and treatments are shown in Tables 1 and 2 and Additional file: Table ST1-ST2. Age-stratified patients (Additional file: Figure SF1) were grouped according to their eventual outcomes at discharge/death into mild, moderate, severe-survivor, and severe-deceased (Tables 1 and 2). A higher proportion of female patients (56%) vs. males (44%) fell into the mild and moderate than severe-survivor outcome groups (Additional file: Figure SF1a. Fisher exact test *p* = 0.0183). Conversely, a higher proportion of male (58%) vs. female (41%) patients fell into the severe-deceased outcome group, albeit without reaching statistical significance (*p* = 0.0511). In both male and female patients, the median age in severe-deceased outcome groups was significantly older than those in mild or moderate outcome groups, as expected [32]. However, women in the severe-deceased group were significantly older than males in the same outcome group (Additional file: Figure SF1c).

**Table 1:** Baseline clinical characteristics of the male study population

	Mild-moderate *N*=114	Severe-recovered *N*=97	Severe-deceased *N*=38	*p*-value^1–2^	*p*-value^1-3^	*p*-value^2–3^
*Age (years), median (IQR)*	59 (56–63)	56 (53–59)	68 (67–71)	0.1096	0.0001	<0.0001
*Length of stay (days), median (IQR)*	8 (7–9)	30.5 (27–34)	19 (9–26)	<0.0001	<0.0001	0.0009
*Comorbidity, n° (%)*	54 (47.36)	57 (58.76)	28 (73.68)	0.1279	0.0051	0.1175
*Hypertension*	35 (30.70)	31 (31.96)	21 (55.26)	0.8822	0.0109	0.0178
*Diabetes*	20 (17.54)	15 (16.46)	11 (28.95)	0.7143	0.1630	0.0908
*Cancer*	6 (5.26)	3 (3.09)	3 (7.89)	0.5115	0.6915	0.3497
*Cardiovascular diseases*	9 (7.89)	12 (12.37)	3 (7.89)	0.3573	0.9999	0.5560
*Chronic Lung diseases*	10 (8.77)	6 (6.19)	5 (13.16)	0.6046	0.5297	0.2912
*Obesity-Dyslipidaemia*	16 (14.03)	33 (34.02)	11 (28.95)	0.0009	0.0497	0.6842
*Chronic Kidney disease*	6 (5.26)	2 (2.06)	1 (2.63)	0.2927	0.6808	0.6727
*Others*	9 (7.89)	13 (13.4)	7 (18.42)	0.2586	0.1220	0.5903
*Biochemical parameters, median (IQR)*						
*Testosterone*	144.3 (112.3–189.3)	52.91 (45.03–66.73)	58.48 (46.83–81.94)	<0.0001	<0.0001	0.9999
*Leucocytes n°*	6.32 (5.78–6.88)	7.80 (6.83–8.80)	8.70 (7.20–9.65)	<0.0001	0.0051	0.9999
*Lymphocytes n°*	1.20 (1.0–1.4)	0.77 (0.70–0.82)	0.8 (0.65–0.90)	<0.0001	0.0001	0.9999
*Lymphocytes %*	18.55 (16.15–1.57)	9.20 (8.18–11.28)	10.89 (7.95–13.38)	<0.0001	<0.0001	0.9999
*Neutrophils*	4.57 (3.92–4.90)	6.26 (5.53–7.38)	6.39 (5.53–8.34)	<0.0001	0.0007	0.9999
*Interleukine-6*	42.30 (25.33–53.46)	130.8 (112.10–160.6)	215.30 (117.6–996.4)	<0.0001	<0.0001	0.9999
*C-reactive protein*	9.57 (6.76–10.88)	17.47 (12.17–0.72)	14.56 (9.84–24.019)	<0.0001	0.0069	0.9999
*LDH*	324 (296.0–369.0)	472.5 (440.0–506.0)	493.0 (429.0–583.0)	<0.0001	<0.0001	0.9999
*D-Dimer*	236 (213.0–311.0)	529.0 (361.0–711.0)	421.5 (260.0–684.0)	<0.0001	0.0210	0.9999
*Ferritin*	934 (679.0–1089.0)	1289 (1100.0–1574.0)	1443.0 (554.9–1919.0)	0.0022	0.7403	0.6783

The Fisher’s exact test was used to compare comorbidities. The Kruskal-Wallis test with Dunn’s multiple comparison was used to analyze the length of stay and biochemical parameters

^1^Mild-moderate group, ^2^Severe-recovered group, ^3^Severe-deceased group

**Table 2:** Baseline clinical characteristics of the female study population

	Mild-moderate *N*=145	Severe-recovered *N*=76	Severe-deceased *N*=27	*p*-value^1–2^	*p*-value^1–3^	*p*-value^2–3^
*Age (years) median (IQR)*	57 (54–60)	55 (53–62)	74 (68–81)	0.9999	<0.0001	<0.0001
*Length of stay (days), median (IQR)*	7 (6–7)	25 (20.0–33.0)	15 (8–19)	<0.0001	0.0033	0.0003
*Comorbidity, n° (%)*	89 (61.38)	47 (61.84)	22 (81.48)	0.9999	0.0503	0.0942
*Hypertension*	43 (29.65)	20 (26.31)	14 (51.85)	0.6408	0.0431	0.0190
*Diabetes*	20 (13.79)	6 (7.89)	2 (7.41)	0.2719	0.5343	>0.9999
*Cancer*	9 (6.21)	5 (6.58)	4 (14.81)	0.9999	0.1259	0.2366
*Cardiovascular diseases*	11 (7.58)	5 (6.58)	3 (11.11)	0.9999	0.4637	0.4290
*Chronic Lung diseases*	12 (8.27)	8 (10.53)	6 (22.22)	0.6249	0.0413	0.1877
*Obesity-Dyslipidaemia*	43 (29.65)	28 (36.84)	8 (29.63)	0.2916	0.9999	0.6396
*Chronic Kidney disease*	5 (3.49)	2 (2.63)	2 (7.41)	0.9999	0.3024	0.2805
*Others*	20 (13.79)	11 (14.47)	5 (18.52)	0.9999	0.5453	0.5461
*Biochemical parameters, median (IQR)*						
*Leucocytes n°*	5.95 (5.40–6.81)	6.28 (5.46–7.02)	7.29 (5.74–9.07)	0.9999	0.2849	0.6427
*Lymphocytes n°*	1.18 (1.04–1.27)	1.00 (0.84–1.12)	0.89 (0.74–1.21)	0.0008	0.0349	0.9999
*Lymphocytes %*	19.76 (17.96–22.16)	15.65 (16.62–18.58)	13.40 (9.59–19.18)	0.0009	0.0025	0.9999
*Neutrophils n°*	4.02 (3.6–4.78)	4.88 (4.23–5.43)	5.83 (4.37–7.66)	0.2125	0.0649	0.9426
*Interleukine-6*	34.21 (26.81–38.60)	75.30 (50.26–86.23)	65.36 (45.03–154.6)	<0.0001	<0.0001	0.9999
*C-reactive protein*	8.38 (4.78–10.44)	12.67 (10.44–14.99)	14.47 (6.43–21.89)	0.0042	0.0846	0.9999
*LDH*	304 (289.0–345.0)	420.0 (380.0–486.0)	415.0 (283.0–539.0)	<0.0001	0.0855	0.9999
*D-Dimer*	258.0 (228.0–275.0)	334.0 (258.0–422.0)	269.0 (207.0–424.0)	0.0379	0.7287	0.9999
*Ferritin*	378.0 (284.0–437.0)	528.0 (445.0–729.0)	445.0 (387.0–730.0)	0.0023	0.3007	0.9999

The Fisher’s exact test was used to compare comorbidities. The Kruskal-Wallis test with Dunn’s multiple comparison was used to analyze the length of stay and biochemical parameters

^1^Mild-moderate group, ^2^Severe-recovered group, ^3^Severe-deceased group

**Fig. 1:**
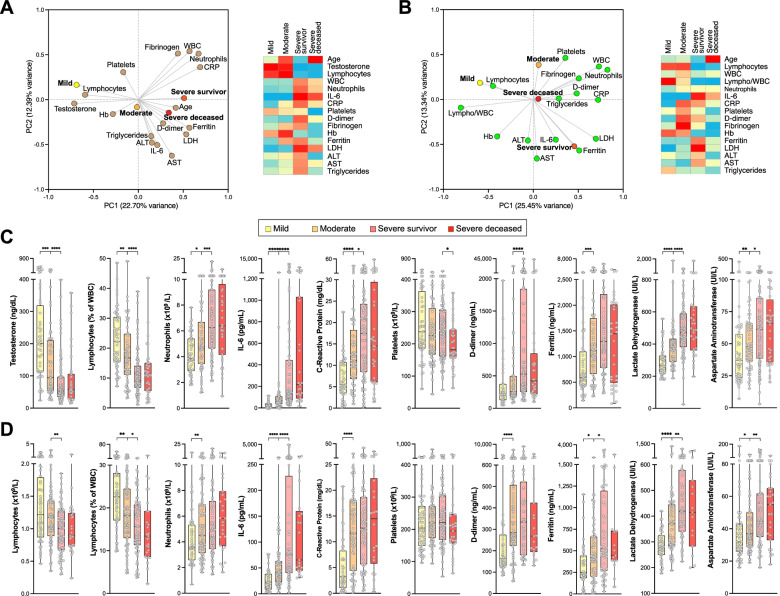
Clinical biochemistry features of male (**A**, **C**) and female (**B**, **D**) COVID-19 patients, associated with outcomes. Clinical biochemistry values were determined for samples collected at patient admission. **A, B** Left panels: Principal component analysis (PCA) illustrating correlations between elevated levels of the indicated parameters and mild, moderate, severe survivor or severe deceased outcomes in male (**A**) or female (**B**) patients. Right panels: Heatmap of correlation coefficients between elevated levels of biochemical parameters and outcomes. Spearman multivariant correlation analyses were performed for all parameters vs. outcomes, the resulting coefficients normalized for each column (range, 0 to 1) and used to build heatmaps. **C**, **D** Values of relevant clinical biochemistry parameters assessed for admission samples and grouped by eventual outcome for male (**C**) and female (**D**) patients. Asterisks denote significance of pairwise comparisons between samples grouped by outcome: *p ≤ 0.05, **p ≤ 0.01, ***p ≤ 0.001, and ****p ≤ 0.0001. Non-significant comparisons (*p* > 0.05) are not shown

As an approach to capture global patterns of association between biochemical parameters and outcomes, we applied principal component analysis (PCA), followed by Spearman multivariate correlation analysis (Fig. 1A, B). In male patients (Fig. 1A), both PCA and multivariate analysis showed a clear correlation between mild or moderate outcomes with known predictors of good outcome, such as higher lymphocyte counts or hemoglobin levels, while severe outcome groups correlated with high neutrophil counts, and high IL-6, CRP, D-dimer, ferritin, or LDH levels, confirming prior evidence [1, 32, 33]. In contrast, patients with moderate or severe outcomes had significantly lower serum testosterone levels (*p* < 0.0001) compared to those with mild outcomes (Table 1, Fig. 1C), in agreement with other studies [12, 13]. Interestingly, the low testosterone levels found in the admission time-point determinations for both severe outcome groups were not significantly different between survivor and deceased patients (Table 1). Furthermore, older age presented a stronger correlation with a severe-deceased outcome than biochemical parameters predictive of poor outcome, such as D-dimer, ferritin, LDH, or IL-6 (Fig. 1A).

In female patients, PCA and multivariate analysis also highlight significant differences between mild-moderate and severe outcomes (Fig. 1B, D). Similar to male patients, the mild outcome group of female patients showed strong correlations to lymphocyte counts and hemoglobin levels, while the severe outcome groups are correlated with high IL-6, CRP, D-dimer, ferritin, and LDH (Fig. 1B, D). Like in male patients, older age showed the strongest correlation to a severe-deceased group in female patients (Fig. 1B; Additional file: Figure SF1c). Age, platelet counts, and fibrinogen levels significantly discriminated severe-survivor from severe-deceased female patients (Fig. 1B, D).

**Fig. 2:**
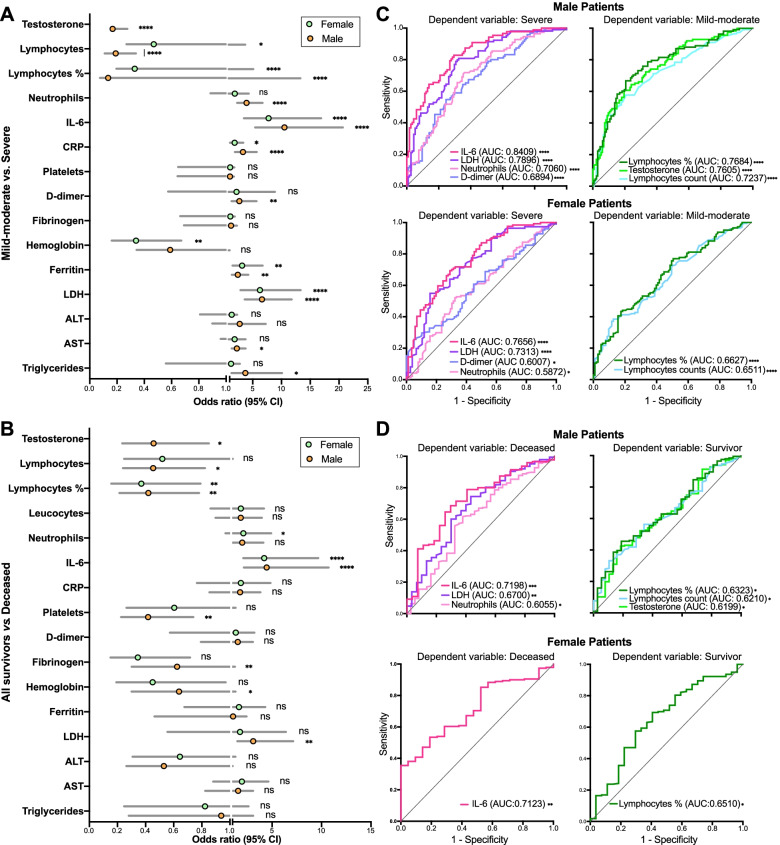
Assessment of clinical biochemistry parameters as predictors of risk of severe disease or death from COVID-19. **A**, **B** Odds ratios (OR) of clinical biochemistry parameters and risk of severe disease (**A**) or death (**B**) in male and female patients. **C**, **D** Receiver operating characteristic (ROC) curves and area under the curve (AUC) values of risk of severe disease (**C**) or death (**D**). Shown are only those parameters with significant AUC values (*p* ≤ 0.05). **E** Correlations of testosterone serum levels with lymphocytes (percentage of WBC and counts) and neutrophil counts

The risk of ICU admission for patients with mild-moderate outcomes was assessed by odds ratio (OR) estimates and logistic regression analysis. In male patients, the most significant OR of ICU admission were found for IL-6 (OR 10.53, 95% CI 5.42 to 20.67), LDH (OR 6.62, 95% CI 3.62 to 11.79), lymphocyte fraction of WBC (OR 0.14, 95% CI 0.08 to 0.25) and neutrophilia (OR 3.95, 95% CI 2.30 to 6.78) (Fig. 2A), in agreement with previous studies [32, 34]. A significant OR was also found for testosterone (0.17, 95% CI 0.09 to 0.31) also in line with other studies [12–14]. In female patients, the most significant OR of ICU admission were for IL-6 (OR 7.77, 95% CI 3.48 to 16.9), LDH (OR 6.26, 95% CI 2.88 to 13.34), ferritin (OR 3.17, 95% CI 1.50 to 6.74), and lymphocyte fraction of WBC (OR 0.33, 95% CI 0.20 to 0.57) (Fig. 2A). Interestingly, testosterone levels on admission were not significantly associated with the occurrence of comorbidities in older men (Additional file: Figure SF2).

The power of these parameters to predict severe disease was corroborated by logistic regression analysis. The resulting receiver operating characteristic (ROC) curves yielded areas under the curve (AUC), which, in male patients, were > 0.7 (*p* < 0.0001) for IL-6, LDH, and neutrophilia, and < 0.23 for lymphopenia and testosterone. In females, AUC of ROC curves were > 0.7 (*p* < 0.0001) for IL-6 and LDH (Fig. 2B).

The same parameters showed a weaker power to predict the risk of death from COVID-19 when comparing all survivors, including severe survivors, versus deceased patients, with the exception of serum IL-6 levels, in both male and female patients (OR 4.45, 95% CI 2.14 to 10.71 and AUC of ROC 0.7189, *p* = 0.0002 for males; and females had OR 4.21 and AUC of ROC 0.7123, *p* = 0.0014) (Fig. 2B, D).

A significant correlation was observed between serum testosterone levels and lymphocyte counts (absolute counts, *r* = 0.3122; fraction of WBC, *r* = 0.4187) and neutrophil counts (*r* = −0.3586), suggesting that these three parameters may be mutually coupled (Fig. 2E).

### Recovery of serum testosterone levels accurately predicts survival in male COVID-19 patients

**Fig. 3:**
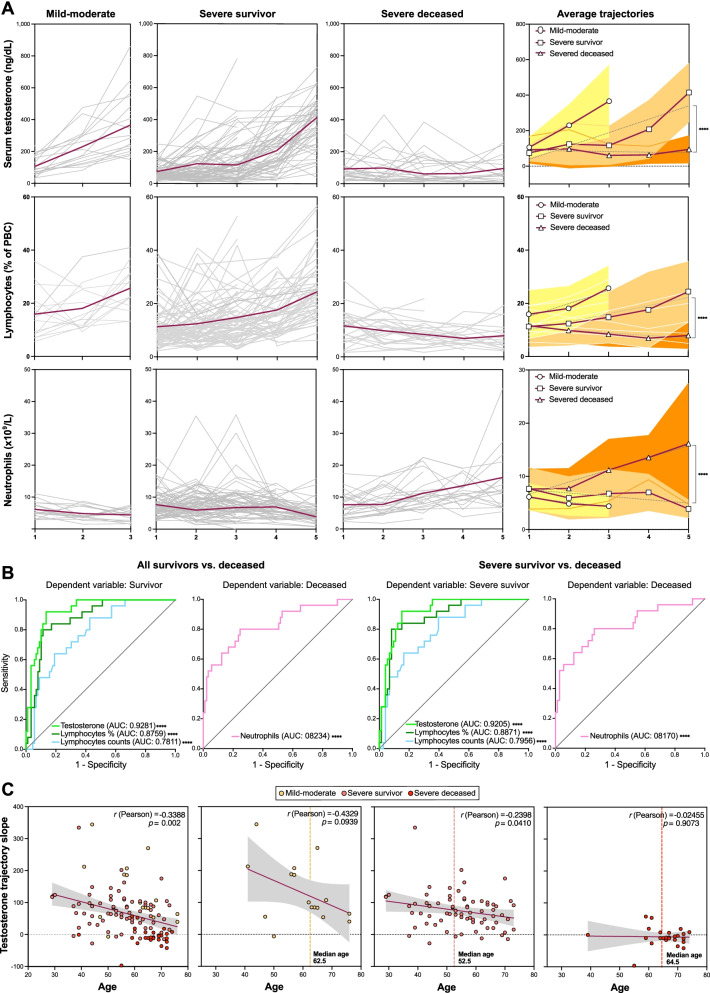
Recovery of serum testosterone levels and blood lymphocyte counts predict survival in male COVID-19 patients. **A** Longitudinal determinations (≥ 3 samples per patient collected on separate dates) of clinical biochemistry parameters were performed, and trajectories for individual patients (grey lines) and average values (red lines) plotted. A given time-point corresponds to a cluster of days post-admission (± 3 days). Linear regression was applied to average trajectories and the resulting slopes compared for significance between outcome groups by means of two-way ANOVA. **B** ROC curves and AUC values for longitudinal trajectories (linear regression slopes) of serum testosterone, blood lymphocyte counts (number per mL and % of white blood cells), and blood neutrophils as predictors of survival in comparisons of all surviving vs. deceased patients (left two panels) or surviving patients with severe disease vs. deceased patients (right two panels). Longitudinal analyses for additional clinical biochemistry parameters are shown in Additional file 1: Figure SF4. **C** Correlations of age with testosterone trajectory slopes in all patients with longitudinal analyses (leftmost panel) and in different outcome groups

In order to explore whether determinations in longitudinal samples could yield additional or improved predictors of lethal disease, we evaluated the trajectories for all clinical biochemical and hematological parameters and plotted for all patients grouped into mild-moderate, severe survivor, and severe deceased outcomes (Fig. 3A and Additional file: Figure SF3). The trajectories of only three parameters, namely testosterone (*p* = 0.0038), lymphocyte counts (or fractions of WBC) (*p* = 0.01), and neutrophil counts (*p* = 0.0023), were significantly different, as analyzed by two-way ANOVA, in comparisons of trajectories between the severe survivor and severe deceased groups (Fig. 3A). No significant differences for any of these variables were observed between severe survivor and mild-moderate outcomes (Fig. 3A). None of the other parameters showed statistically significant different trajectories in comparisons between severe survivor vs. severe deceased outcomes (Additional file: Figure SF3).

The resulting ROC curves and AUC values, calculated from the slopes of the trajectories, indicated that serum testosterone trajectories are remarkably accurate predictors of survival from COVID-19, both in all-survivor vs. deceased (AUC = 0.9281, 95% CI 0.8801 to 0.9761, *p* < 0.0001) and severe survivor vs. deceased (AUC = 0.9205, 95% CI 0.8664 to 0.9747, *p* < 0.0001) outcome comparisons (Fig. 3B). Lymphocyte counts (numbers per dL or fractions of WBCs) were also highly accurate predictors of outcome, as were neutrophil counts (Fig. 3B). Interestingly, the trajectories of IL-6 or LDH, whose values on admission were predictive of severity and death from COVID-19 in male patients, were not significantly different in these longitudinal comparisons (Additional file: Figure SF3). These results suggest a role for testosterone in deregulation of the immune response in deceased patients, in particular for the observed lymphopenia and neutrophilia.

Age is a predictor of COVID-19 severity [32]. In our cohort of male patients, the testosterone trajectory slopes significantly and inversely correlated with age (*r* = −0.3801, *p* < 0.0001) (Fig. 3C). Consistently, a majority of patients with severe deceased outcomes had low or negative testosterone trajectory slopes (Fig. 3C). However, and interestingly, the median age of patients with severe deceased outcomes was not significantly different from the median age of patients with mild-moderate outcomes, and a substantial proportion of patients with severe survivor outcomes were aged older than 60 (Fig. 3C). Likewise, while the frequency of comorbidities was higher among patients with fatal outcomes as compared with those who survived severe disease, it was not significantly different from the frequency of the moderate outcome group (Additional file: Table 1, Figure SF1). Similar to total circulating testosterone, free testosterone levels were significantly decreased in severe patients in comparison to the mild-moderate group (Additional file 1: Figure SF4). In addition, serum levels of sex hormone-binding globulin (SHBG), the most abundant circulating testosterone binding protein, showed a significant association of with older age in mild-moderate patients (*p* = 0.0237), but not in severe patients*.*

These observations suggests that old age, with or without accompanying comorbidities, may impact the ability of a subset of COVID-19 patients to reinstate testosterone production, coupled to a failure to recover from the disease.

### The LH-androstenedione axis is not significantly perturbed in male COVID-19 patients

**Fig. 4:**
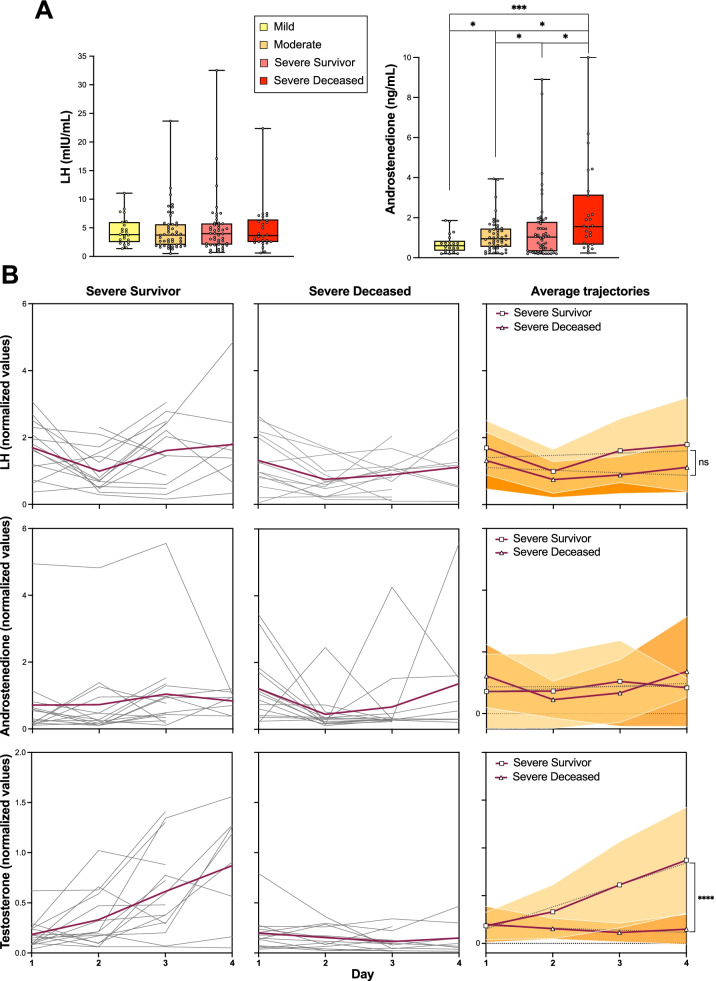
The luteinizing hormone (LH)-androstenedione axis is not significantly perturbed in male COVID-19 patients. **A** Determinations of serum LH and androstenedione levels in samples collected at admission, grouped by eventual outcomes. Pair-way between-group comparisons were performed by *t* test. **B** Longitudinal determinations (≥ 3 samples) of serum LH, androstenedione, and testosterone levels, analyzed as in Fig. 3. Comparisons of trajectories (linear regression slopes) were performed by two-way ANOVA

Testosterone is synthesized from androstenedione in the Leydig cells of the testis under the stimulus of luteinizing hormone (LH), secreted from the anterior portion of the pituitary gland. The observed critical decline in circulating testosterone levels in male COVID-19 patients suggests the occurrence of a transient (survivor outcomes) or sustained (fatal outcomes) hypogonadism following the onset of COVID-19. To address potential mechanisms explaining the observed failure to recover circulating testosterone levels in fatal COVID-19, we determined circulating LH and androstenedione levels in a longitudinal series of samples in a patient subcohort for which the testosterone trajectories had been concomitantly determined. The median levels of LH fell within normal ranges, independent of patient outcome (Fig. 4A). Similarly, longitudinal LH trajectories were not significantly different between patients in the survivor vs. deceased outcomes, in stark contrast with the strongly divergent testosterone trajectories (Fig. 4B). Nevertheless, LH levels determined in the last of the longitudinal samples showed a decline in the deceased outcome group as compared to the severe survivor group, although without reaching the statistical significance. On the other hand, although androstenedione levels fell within normal ranges in the majority of patients in all outcome groups and throughout the longitudinal analysis (Fig. 4A, B), deceased patients showed increased levels as compared to the survivor outcome groups, without a concomitant increase in testosterone levels (Fig. 4A). As such, the failure to recover physiological levels of testosterone in patients with fatal outcomes, in spite of LH and androstenedione levels within normal ranges, and the lack of rise in LH expected with low circulating testosterone, suggest the development of a combined central and peripheral (Leydig cell failure) malfunction in the biosynthesis of testosterone in these patients.

### Lethal male COVID-19 is associated with a depletion of circulating T helper cells

**Fig. 5:**
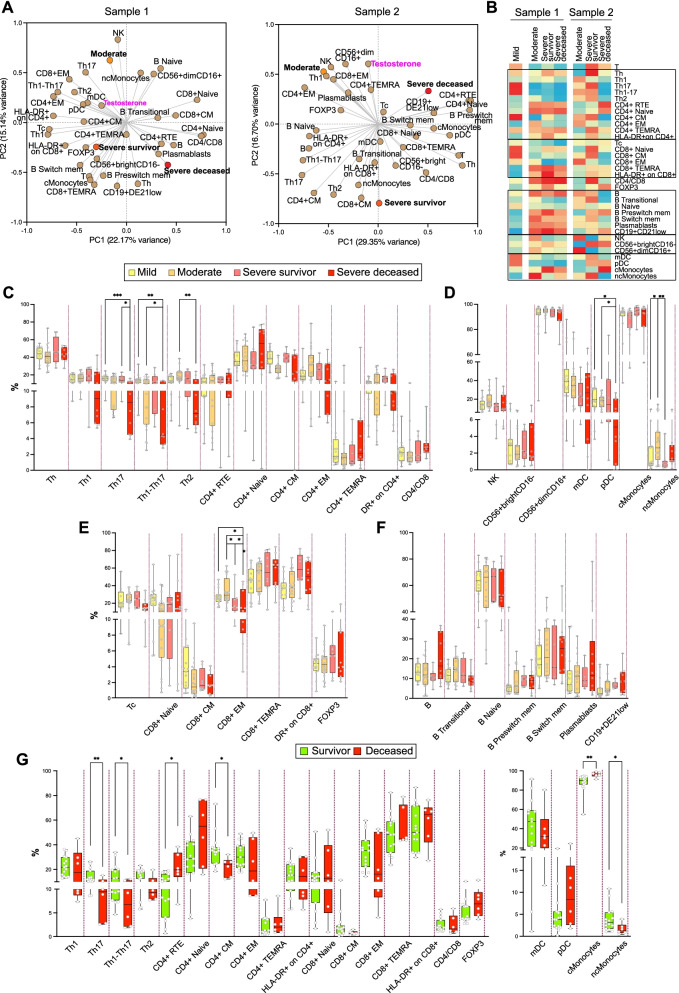
Immune switch during the course of disease in severe and deceased patients, as determined by multiparameter profiling of circulating immune cells. **A** PCA of samples analyzed near admission (Sample 1, left panel) and near discharge or death (Sample 2, right panel). Mild, moderate, severe survivor, and severe deceased outcomes were assigned values 1, 2, 3, and 4, respectively. Serum testosterone values of samples collected in the same or nearby dates (± 3 days) were included in the analysis. The indicated immune subpopulations are defined by cell-surface markers and determined by spectral flow cytometry (Materials and methods). **B** Heatmap of correlation coefficients between immune subpopulation values and outcomes, for near-admission (Sample 1, left Heatmap) and near-discharge/death (Sample 2, right Heatmap) samples. Spearman multivariant correlation analyses were performed for all parameters vs. outcomes, the resulting coefficients normalized for each column (range, 0 to 1) and used to build heatmaps. **C**–**F** Between-outcome comparisons of immune cell subpopulation: CD4+ (**C**); natural killer, dendritic, and monocyte (**D**); CD8+ (**E**); and B (**F**) cell subpopulations. **G** Survivor (mild, moderate, severe survivor) vs. deceased patient comparisons for T cell (CD4+ and CD8+) and dendritic cells and monocytes. Such comparisons were not significant for other immune subpopulations (B cells, NK cells)

A number of studies have found substantial differences in immune responses to SARS-CoV-2 between male and female patients [16, 18], although the mechanisms underlying these differences are still unclear. In order to address the relationship between testosterone trajectories, outcome, and immune status in male patients, we analyzed circulating immune subpopulation repertoires in a subset of our patient cohort, in at least two independent determinations, separated by 5–20 days. These analyses point to a correlation between immune subpopulations, outcome, and testosterone levels both in a first determination of samples near admission date (Sample 1) and a subsequent analysis of samples near discharge or death (Sample 2), with some subpopulations showing remarkable shifts between Sample 1 and Sample 2 in their correlations with outcome (Fig. 5A, B; Additional file: Figure SF5). As such, Sample 1 determinations demonstrated relatively few changes in immune cell repertoires between surviving and deceased patients. In stark contrast, a subsequent determination (Sample 2) showed a coordinated depletion of T helper subpopulations in association with death, along with changes in natural killer (CD56^+bright^CD16^-^ and CD56^+dim^CD16^+^) and monocyte subpopulations (Fig. 5A). The subpopulations with the most significant changes in relative abundance as a function of outcome tended to correlate with serum testosterone levels sampled in the same period (1–3 days from sampling for immune repertoire analyses) (Fig. 5A).

These correlations were more evident in multivariate correlation analyses, particularly for Sample 2 (Fig. 5B). These analyses show a generalized loss of representation of circulating differentiated and polarized T helper subpopulations in deceased patients (Th1, Th17, Th1-Th17, Th2, central memory CD4^+^, effector memory CD4^+^, CD4^+^ TEMRA) compared to surviving patients, accompanied with a reciprocal increased representation of undifferentiated CD4+ cells (recent thymus emigrant CD4^+^, naïve CD4^+^) (Fig. 5B–D). They also point to an association of monocyte differentiation with outcome, with a predominant correlation with non-classical monocytes in moderate and severe survivor patients and, conversely, with significantly more pro-inflammatory [35] classical monocytes in deceased patients (Fig. 5B–D). There are additional associations of cytotoxic T cell or B cell subpopulations with lethal COVID-19 (Fig. 5D–F), albeit without reaching statistical significance. These correlations were made more evident when comparing all surviving patients (moderate and severe survivor) with deceased patients (Fig. 5G) and further illustrated by flow cytometry histograms of representative cases (Additional file: Figure SF5).

Together, these observations indicate that male COVID-19 patients with a lethal outcome suffer from a decline in circulating differentiated T helper cells accompanied with a relative enrichment in circulating undifferentiated CD4^+^ T cells.

## Discussion

Numerous studies have identified prognostic markers able to discern severe COVID-19 patients, including older age, male sex, co-morbidities such as obesity, diabetes, or cardiovascular disease, elevated circulating markers of inflammation, lymphopenia, neutrophilila [2, 12, 17, 32–34, 36], or the presence of autoantibodies to class-I interferons [37]. Nomograms or scores that combine several independent parameters have been proposed as predictors of COVID-19 outcome [2, 12, 32]. However, relatively few studies have addressed sex differences in predictive markers of disease outcome [2, 12, 32, 38–40]. Interestingly, inflammation markers, but not co-morbidities, BMI, or age, have been found to be associated with outcome differences between male and female COVID-19 patients [40].

Our comparative analysis of biochemical and hematological parameters has revealed that both sexes share markers with significant predictive power of disease outcome, including IL-6, LDH, D-dimer, lymphopenia, and neutrophilia. Nevertheless, the levels of these markers, and the strength of their predictive power, are consistently higher in male patients as compared to female patients. This becomes more evident when evaluating predictive markers of lethal COVID-19, which yields IL-6 and lymphocyte (percentage of total WBC) as the only two significantly predictive markers shared in both male and female patients. Other significant markers predictive of lethal COVID-19 in male but not female patients are LDH levels, neutrophilia, and absolute lymphocyte counts, in addition to testosterone levels, which are exclusively masculine in our patient cohorts. Furthermore, we found significant and direct correlations between testosterone levels, lymphocytes, and neutrophils, suggesting a role for testosterone in aberrant immune responses in deceased patients.

These observations suggest that male COVID-19 patients with severe and lethal disease suffer from more deleterious underlying pathogenic and inflammatory processes than female patients with comparable clinical severity [2], a situation also observed in other respiratory viral infections [4, 5]. Our baseline analysis reveals that critically low serum testosterone levels in male patients are a risk factor for severe COVID-19, along with other factors predictive of severity that are in line with prior evidence [32, 34]. We have also found that male COVID-19 patients with a higher risk of progression to a severe-critical disease present higher levels of inflammatory markers (serum IL-6, blood neutrophil counts) and tissue damage (LDH), and more marked lymphopenia [2], as compared to age-matched female patients.

Interestingly, markers of inflammation (IL-6, CRP) or tissue damage (LDH), with good outcome predictive power in admission sample determinations, lost their predictive power in longitudinal analyses in male patients, collected up to the time of discharge or death. In contrast, testosterone levels, whose determinations on admission provided a relatively modest outcome predictive power, corroborating other studies [11, 13], gained remarkable levels of significance when analyzed longitudinally. The AUC values of ROC curves in logistic regression analyses (mild-moderate vs. severe: 0.9281, 95% CI 0.7216 to 0.9252, *p* < 0.0001; severe survivor vs. deceased: 0.9205, 95% CI 0.8664 to 0.9747, *p* < 0.0001) indicate that serum testosterone trajectories in longitudinal determinations constitute, to the best of our knowledge, the most accurate independent predictors of disease outcome in male COVID-19 patients described thus far. Furthermore, longitudinal trajectories of lymphocyte and neutrophil counts also yield highly significant predictions of disease outcome.

Other biochemical parameters indicative of pathological inflammatory or pro-coagulant states, such as elevated IL-6, CRP or D-dimer levels, eventually return to near-physiological levels in both survivors and patients with fatal outcomes. This has been observed in other studies [40, 41] and suggests that the normalization of these factors is insufficient, per se, to abate the pathological hyperinflammation and hypercoagulation accompanying severe COVID-19 with a fatal outcome, which might require the concomitant alleviation of lymphopenia and neutrophilia. On the other hand, although different stimuli and conditions such as mechanical ventilation, muscle immobilization, severe sepsis, and multiple organ dysfunction as well as neuro/myotoxic agents may contribute to a critical status among patients admitted to ICU [42], all severe patients in our study, with either survivor or deceased outcomes, were under comparable pharmacological and physical management (Additional file 1: Tables ST1 and ST2), and thus, these factors are unlikely to contribute to the differential outcomes in this study.

We have found that testosterone trajectories are not paralleled by changes in circulating LH or androstenedione trajectories expected in the presence of functioning physiological feedback loops. This could be explained by an inhibition of the LH-androstenedione axis, which has been associated with non-specific critical illness [43] and the deleterious action on the hypophysis of inflammatory cytokines [44]. A second possible mechanism may involve infection and damage by SARS-CoV-2 of ACE2-expressing testicular cells, mainly Leydig cells [45, 46]. In the first scenario, acute declines in LH and adrostenedione levels would be expected, while in the latter scenario, they would be either unaffected or increased for LH due to a negative feedback loop with testosterone [43]. However, we observed that LH levels showed a slight decline in deceased patients, while androstenedione levels were increased. It should be noted that circulating androstenedione is produced mostly by adrenal glands [47] and its synthesis might be affected by the corticosteroids used to treat these patients (Additional file: Table ST1B), which may block the endogenous production of cortisol, corticosterone, and aldosterone [47]. Therefore, a likely mechanism to explain the failure of patients with fatal outcomes to recover their physiological levels of testosterone, combined with normal androstenedione levels and a lack of rise in LH, suggests a malfunction of the testosterone-LH feedback loop. As such, an irreversible damage of Leydig cells [48] in patients with fatal outcomes could explain these observations, while a resolution of viral infection would explain the recovery of a normal production of testosterone in survivors.

Another relevant factor associated with late-onset hypogonadism [49], as well as with an irreversible failure to reinstate testosterone production after critical situations that may compromise the LH-androstenedione axis, is old age [50], which has been linked to senescent dysfunction of Leydig cells [51]. The fact that a majority of non-survivor patients in our study who failed to reinstate testosterone levels are older than 60 years of age would be consistent with the senescence hypothesis. However, our cohort has more patients older than 60 who reinstated their testosterone levels and survived severe COVID-19. Therefore, either Leydig cell senescence only affects a small subset of older patients or other mechanisms may be invoked to explain failure to restore testosterone production.

Conversely, these observations also suggest that while the reinstatement of physiological testosterone levels may be mechanistically linked to a return to lymphocyte and neutrophil homeostasis, it may not be required for the relative normalization of other inflammatory pathways, arguably driven by an unmitigated production of IL-6 and other pro-inflammatory cytokines triggered by acute viral infection [52]. Our observations suggest that sufficient and timely resolution of pathogenic hyperinflammation to prevent a lethal outcome may require the additional return to homeostasis of innate and/or adaptive immune cell dynamics and function, possibly assisted in male patients by the reinstatement of testosterone production.

There is now a wealth of studies describing the dynamics of immune responses to acute and subacute infection with SARS-CoV-2, including multiparameter and functional analyses of circulating and tissue-associated innate and adaptive immune subpopulations [53, 54]. Some of these studies have addressed sex differences in such responses [11, 14]. Our analysis in male patients indicates an association of specific immune subpopulations with COVID-19 outcome and a shift of such associations from early (Sample 1) to late (Sample 2) time-points in the course of the disease. For example, the relative representation of differentiated (CD8^+^ TEMRA, CD4^+^ TEMRA) and activated (HLA-DR^+^ on CD4^+^ and on CD8^+^) T cell subpopulations and differentiating B cells modestly correlated with all outcomes except mild disease in Sample 1. This indicates an ongoing early immune response of similar nature and magnitude, regardless of final outcome, as reported by others [53]. In this phase, non-classical monocytes are more prevalent than classical monocytes in patients with a moderate outcome, while patients with severe survivor and severe deceased outcomes show a predominance of more inflammatory [55] classical monocytes, in support of a more inflammatory state of these patients, as also evidenced by the clinical biochemical and hematological parameters discussed above.

However, later in the course of disease (Sample 2), a remarkable shift takes place, in particular with regard to correlations with severe survivor as compared to severe deceased patients. As such, while patients with severe survivor outcomes show positive correlations with differentiated (CD4^+^ TEMRA, CD8^+^ TEMRA), activated (HLA-DR^+^ on CD4^+^ and on CD8^+^), and memory (CD4^+^ central memory, CD8^+^ central memory) T cell subpopulations, patients with eventual fatal outcomes evidence a depletion of these subpopulations, along with an accumulation of undifferentiated T helper cells (recent thymic emigrant CD4^+^ and naïve CD4^+^). This late shift also affects innate immune populations, as severe survivor patients correlate with non-classical monocytes over classical monocytes, while the reverse is the case for severe deceased patients. Similar observations have been made by others in studies correlating innate [53] and adaptive [17] immune cell subpopulations to COVID-19 outcome.

Importantly, our study additionally correlates relative representations of immune subpopulations to testosterone levels. Thus, higher testosterone levels in Sample 1 are correlated to polarized (Th1, Th17, Th1-17, Th2) and differentiated (effector memory CD4^+^, central memory CD4^+^, CD8^+^ TEMRA) T cell subpopulations. In Sample 2, testosterone levels correlate to a similar range of subpopulations, along with plasmablasts and mature NK cells (CD16^+dim^CD16^+^). These temporal switches in the differentiation profiles of distinct immune subpopulations may suggest that in patients with lethal outcomes, there may be a defective differentiation of T helper cells [56] and monocytes. A second possible explanation of the apparent depletion of circulating differentiated and polarized cells may be an enhanced clearance or migration from circulation to peripheral tissues [57]. Finally, specific subpopulations may become exhausted in late stages of the disease [58–60]. These three putative mechanisms are not mutually exclusive and may take place either simultaneously or dynamically at different time points along the clinical course of the patients. Notably, very recent studies recognize T cell apoptosis and depletion as a feature defining severe COVID-19 [60].

The observed concordance of lethal outcome in male COVID-19 patients with (i) persistent lymphopenia and neutrophilia, (ii) depletion of circulating differentiated T helper and T cytotoxic cells and non-classical monocytes, (iii) accumulation of undifferentiated immune counterparts, and (iv) failure to reinstate physiological levels of testosterone, mirrored by converse phenotypes in severe survivor patients who have undergone equivalent critical illness and management, makes it appealing to hypothesize a mechanistic relationship bonding these coincident phenotypes. Relevantly, sex hormones have a profound influence on innate and adaptive immune system development, differentiation, and response to challenge [14, 18] More specifically, androgens have a global anti-inflammatory effect [26, 61], reflected in higher frequencies of autoimmune diseases in women or in acquired or genetically determined hypogonadism [21], as compared to men with a normal XY chromosome complement. On the other hand, testosterone replacement therapy in hypogonadal men attenuates inflammation [62] and androgens suppress thymic precursor development [63] and promote the terminal differentiation of T cell subpopulations [64] and monocyte precursors [65]. Conversely, androgen deprivation through surgical or pharmacological castration in animal models prompts the regeneration of the thymus in aged mice, leading to a relative accumulation of undifferentiated T cell populations (RTE and naïve T cells) [66] and classical monocytes [67]. A similar effect of androgen deprivation on T cell development and differentiation has been observed in prostate cancer patients, with an expansion of RTE and naive T cells, particularly among CD4^+^ cells [66, 68].

## Conclusions

The tight association observed between reinstatement of testosterone and survival from COVID-19 in male patients, along with a reversal of signs of excessive inflammation and immune dysfunction, suggests a potential functional role for testosterone, beyond being a mere biomarker of outcome, in such recovery. Further explorations of mechanistic relationships between testosterone status and SARS-CoV-2 infection outcomes may lead to potential prophylactic or therapeutic interventions to tackle severe and lethal COVID-19 in men.

### Study limitations

The limitations of our study include its observational nature on retrospective patients and samples, which has hampered the collection of samples at precisely equivalent time points after symptom onset for all patients. Notably, the retrospective analyses impacted our longitudinal studies of immune populations which, in the present study, were limited to a relative small number and two temporally separate determinations per male patient studied. Similarly, the retrospective nature of our study has precluded assessing the potential impact of the circadian rhythm of testosterone production in our patients. A further limitation was the determination of testosterone by immunoassay and not by gold-standard mass spectrometry (not available in the clinical setting)*.* On the other hand, several hypotheses laid out here would require formal testing by means of approaches that are not addressed in the present study. For example, the various mechanisms postulated to explain the depletion of circulating differentiated T cells in lethal COVID-19 patients would benefit from additional analyses of senescent and activation states of such populations with appropriate markers, as well as detailed analyses of viral infection and immune cell populations infiltrating key tissues, mainly the lung and testis. Finally, pre-clinical animal models would be required for a robust experimental demonstration of mechanistic relationships between testosterone status (e.g., deprivation and replacement) and SARS-CoV-2 infection outcomes, which should also contemplate factors such as age.

## Supplementary Information

**Additional file 1: Table ST1.** Treatments comparison by outcome in male patients. **Table ST2.** Treatments comparison by outcome in female patients. **Table ST3.** WHO classification of disease outcome. **Table ST4.** Panels and antibodies used for immunophenotyping. **Figure SF1.** Patients distribution by outcome, age, and comorbidities. **Figure SF2.** Distribution of male patients with comorbidities according to age and testosterone levels. **Figure SF3.** Longitudinal analysis of serum levels of IL-6, C-reactive protein (CRP), ferritin and lactate dehydrogenase (LDH) in male patients. **Figure SF4.** Bioavailable testosterone serum levels and correlation between age and sex-hormone binding globulin (SHBG). **Figure SF5.** Flow cytometry analysis of circulating immune subpopulations in three illustrative cases with moderate, severe survivor and severe deceased outcomes.

## Data Availability

The datasets used and/or analyzed during the current study are available from the corresponding author on reasonable request.

## Abbreviations

ACE2: Angiotensin-converting enzyme 2
AR: Androgen receptor
AUC: Area under the curve
BMI: Body mass index
COPD: Chronic obstructive pulmonary disease
DC: Dendritic cells
FDA: Food and Drug Administration
ICU: Intensive care unit
IL-6: Interleukin-6
ILC2: Group 2 innate lymphoid cells
LDH: Lactate dehydrogenase
LH: Luteinizing hormone
MERS-CoV: Middle East respiratory syndrome
NK: Natural killer cells
PCA: Principal component analysis
PCR: C-reactive protein
RTEs: Recent thymic emigrant cells
ROC: Receiver operating characteristic
SARS-CoV: Severe acute respiratory syndrome-coronavirus
TEM: T cells effector memory
TEMRA: T cell terminal effector memory
Th: T helper cells
TMPRSS2: Transmembrane protease serine 2
Treg: T regulatory cells
WBC: White blood cells
