# Supplementary material for: Recovery of serum testosterone levels is an accurate predictor of survival from COVID-19 in male patients
Source: BMC Med. 2022 Mar 29;20:129. doi: 10.1186/s12916-022-02345-w (PMC8963401; doi:10.1186/s12916-022-02345-w)
Supplement: Supplementary file 1 — Additional file 1: Table ST1. Treatments comparison by outcome in male patients. Table ST2. Treatments comparison by outcome in female patients. Table ST3. WHO classification of disease outcome. Table ST4. Panels and antibodies used for immunophenotyping. Figure SF1. Patients distribution by outcome, age, and comorbidities. Figure SF2. Distribution of male patients with comorbidities according to age and testosterone levels. Figure SF3. Longitudinal analysis of serum levels of IL-6, C-reactive protein (CRP), ferritin and lactate dehydrogenase (LDH) in male patients. Figure SF4. Bioavailable testosterone serum levels and correlation between age and sex-hormone binding globulin (SHBG). Figure SF5. Flow cytometry analysis of circulating immune subpopulations in three illustrative cases with moderate, severe survivor and severe deceased outcomes. [file 12916_2022_2345_MOESM1_ESM.docx]

**Additional File**

**Recovery of serum testosterone levels is an accurate predictor**

**of survival from COVID-19 in male patients**

Emily Toscano-Guerra^1,2, 11^, Mónica Martínez Gallo^3,4,5^*, Iria Arrese-Muñoz^3,4,5^, Anna Giné^1,2^, Noelia Díaz-Troyano^1^, Pablo Gabriel-Medina^1^, Mar Riveiro-Barciela^6^, Moisés Labrador-Horrillo^6^, Fernando Martinez-Valle^6^, Adrián Sánchez-Montalvá^7^, Manuel Hernández-González^3,4,5^, Ricardo Pujol Borrell^3,4,5^, Francisco Rodríguez-Frias^1^, Roser Ferrer^1^, Timothy M. Thomson^8,9,10, 11^*, and Rosanna Paciucci^1,2^*

*^1^ Biochemistry Service, Vall d’Hebron Hospital, Autonomous University of Barcelona (UAB), Barcelona, Spain*

*^2^ Cell Signaling and Cancer Progression Laboratory, Vall d’Hebron Institute of Research (VHIR), Barcelona, Spain*

*^3^ Immunology Division, Vall d’Hebron Hospital, Barcelona, Spain*

*^4^ Diagnostic Immunology Research Group, Vall d’Hebron Research Institute (VHIR), Barcelona, Spain*

*^5^ Department of Cell Biology, Physiology and Immunology, Autonomous University of Barcelona (UAB), Barcelona, Spain*

*^6^ Internal Medicine Service, Vall d’Hebron Hospital, Barcelona, Spain*

*^7^ Infectious Diseases Department. International Health and Tuberculosis Unit National Referral Centre for Tropical Diseases, Vall d'Hebron University Hospital, Vall d'Hebron Institute of Research (VHIR), Barcelona, Spain*

*^8^ Barcelona Institute for Molecular Biology, National Science Council (IBMB-CSIC), Barcelona, Spain*

*^9^ Networked Center for Hepatic and Digestive Diseases (CIBER-EHD), Instituto Nacional de la Salud Carlos III, Madrid, Spain*

*^10^ Plataforma Temática Interdisciplinar Salud Global (PTI-Global Health) CSIC, Spain*

*^11^ Universidad Peruana Cayetano Heredia, Lima, Perú.*

**Table ST1. Treatment comparison by outcome in male patients.**

| 1. Comparison between Mild-moderate and Severe outcomes | | | | | | |
| --- | --- | --- | --- | --- | --- | --- |
| Treatments | Total  249 (%) | Mild-Moderate  114 (%) | | Severe  135 (%) | | p-value* |
| Hydroxychloroquine | 241 (96.79) | | 112 (98.24) | 129 (95.55) | 0.2953 | |
| Antibiotics | 238 (95.58) | | 104 (91.23) | 134 (99.26) | 0.0031 | |
| Antivirals | 196 (78.71) | | 93 (81.58) | 103 (76.30) | 0.3528 | |
| Corticoids | 66 (26.5) | | 13 (11.40) | 53 (39.26) | <0.0001 | |
| Immunomodulators | 130 (52.21) | | 23 (20.17) | 107 (79.26) | <0.0001 | |
| Anticoagulants | 123 (49.40) | | 34 (29.82) | 89 (65.92) | <0.0001 | |
| Analgesics | 28 (11.24) | | 13 (11.40) | 15 (11.11) | 0.9999 | |

| 1. Comparison between Severe-survivor and Severe-deceased | | | | | |
| --- | --- | --- | --- | --- | --- |
| Treatments | Severe-survivor  97 (%) | Severe-deceased  38 (%) | | p-value* | |
| Hydroxychloroquine | 95 (97.93) | | 34 (89.47) | 0.0526 |  |
| Antibiotics | 96 (98.97) | | 38 (100.0) | 0.9999 |  |
| Antivirals | 76 (78.35) | | 27 (71.05) | 0.3764 |  |
| Corticoids | 33 (34.02) | | 20 (52.63) | 0.0525 |  |
| Immunomodulators | 76 (78.35) | | 31 (81.57) | 0.8149 |  |
| Anticoagulants | 68 (70.10) | | 21 (55.26) | 0.1108 |  |
| Analgesics | 13 (13.40) | | 2 (5.26) | 0.2322 |  |

**Table ST2.** **Treatment comparison by outcome in female patients.**

| Treatments | Total  248 (%) | Mild-Moderate  145 (%) | Severe  103 (%) | P-value* |
| --- | --- | --- | --- | --- |
| Hydroxychloroquine | 228 (91.94) | 133 (91.72) | 95 (92.23) | 0.9999 |
| Antibiotics | 231 (93.15) | 128 (88.28) | 103 (100.00) | 0.0001 |
| Antivirals | 215 (86.69) | 129 (88.97) | 86 (83.5) | 0.2557 |
| Corticosteroids | 41 (16.53) | 11 (7.59) | 30 (29.13) | <0.0001 |
| Immunomodulators | 95 (38.31) | 23 (15.86) | 72 (69.90) | <0.0001 |
| Anticoagulants | 70 (28.23) | 10 (6.90) | 60 (58.25) | <0.0001 |
| Analgesics | 65 (26.21) | 40 (27.59) | 25 (24.27) | 0.6605 |

*Two-by-two Contingency analyses were performed to compare outcomes in each treatment. *P*-values are from Fisher exact test.

| Table ST3. WHO classification of disease outcome (adapted from Grain et al.^26^) | | | |
| --- | --- | --- | --- |
| Group | Outcome | Definition | Stay Type |
| 1 | Mild | Not hospitalized or hospitalized without oxygen | Discharge from emergency to home or Ward |
| 2 | Moderate | Hospitalized with low flow oxygen by mask or nasal prongs or with high flow oxygen | Ward |
| 3 | Severe-Survivor | Hospitalized with non-invasive ventilation or with invasive mechanical ventilation | ICU |
| 4 | Severe-Deceased | Death | Exitus |

**Table ST4.** **Panels and antibodies used for immunophenotyping.**

| **General lymphocyte populations** | **Fluorophore** | **Isotype** | **Clone** |
| --- | --- | --- | --- |
| CD45/CD8/CD4/CD3 | FITC/PE/ECD/PC5 | IgG2b/IgG1/IgG1/IgG1 | B3821F4A/SFCI12T4D11/SFCI21Thy2D3/UCHT1 |
| CD45/CD56/CD19/CD3 | FITC/PE/EDC/PC5 | IgG2b/IgG1/IgG1/IgG1 | B3821F4A/SFCI12T4D11/SFCI21Thy2D3/UCHT1 |
|  |  |  |  |
| **T-cell populations** |  |  |  |
| CXCR3/CD183 | AF488 | IgG1 | G025H7 |
| CCR7/CD197 | PE | IgG2a | G043H7 |
| CD45RA | ECD | IgG1 | ALB11 |
| CCR6/CD196 | PC7 | IgG2a | B-R35 |
| CD4 | APC | IgG1 | 13B8.2 |
| CD8 | APC700 | IgG1 | SFCI21Thy2D3 (T8) |
| CD3 | APC750 | IgG1 | UCHT1 |
| HLA-DR | PB | IgG1 | Immu-357 |
| CD45 | KRO | IgG1 | J.33 |
|  |  |  |  |
| **Recent Thymic Emigrant** |  |  |  |
| CD31 | FITC | IgG1 | 5.6E |
| CD62L | PE | IgG1 | DREG56 |
| CD3 | ECD | IgG1 | UCHT1 |
| CD27 | PC7 | IgG1 | 1A4CD27 |
| CD4 | APC | IgG1 | 13B8.2 |
| CD45RA | PB | IgG1 | 2H4LDH11LDB9 (2H4) |
| CD45 | KRO | IgG1 | J.33 |
|  |  |  |  |
| **T regulatory cell population** |  |  |  |
| CD45RO | FITC | IgG2a | UCHL1 |
| CD25 | PE | IgG2a | B1.49.9 |
| CD3 | ECD | IgG1 | UCHT1 |
| CCR4/ CD194 | PC7 | IgG1 | 1G1 |
| CD4 | APC | IgG1 | 13B8.2 |
| CD127 | APC700 | IgG1 | R34.34 |
| HLA-DR | PB | IgG1 | Immu-357 |
| CD45 | KRO | IgG1 | J.33 |
|  |  |  |  |
| **DC/Monocytes/NK** |  |  |  |
| CD16 | FITC | IgG1 | 3G8 |
| CD11c | PE | IgG1 | BU15 |
| CD3 | ECD | IgG1 | UCHT1 |
| CD19 | ECD | IgG1 | J3-119 |
| CD20 | ECD | IgG2a | B9E9(HRC20) |
| CD56 | PC7 | IgG1 | N901 (NKH-1) |
| CD123 | APC | IgG1 | SSDCLY107D2 |
| CD14 | APC750 | IgG1 | RMO52 |
| HLA-DR | PB | IgG1 | Immu-357 |
|  |  |  |  |
| **B-cell populations** |  |  |  |
| IgD | FITC | IgG2a | IA6-2 |
| CD21 | PE | IgG1 | BL13 |
| CD19 | ECD | IgG1 | J3.119 |
| CD27 | PC7 | IgG1 | 1A4CD27 |
| CD24 | APC | IgG1 | ALB9 |
| CD38 | APC750 | IgG1 | LS198-4-3 |
| IgM | PB | IgG1 | SA-DA4 |
| CD45 | KRO | IgG1 | J.33 |

**Figure SF1 (linked to Tables 1 and 2)**. Patient distributions by (**a**) outcome: Mild-moderate (n = 259), severe-survivor (n =173) and severe-deceased (n = 65). Two by two Contingency analyses shows more proportion of female patients in mild-moderate than severe-survivor outcome (Fisher exact test *p*= 0.0183) **b**), age, (**c**) age and outcome (**d**) and comorbidities and outcome. For 1c, unpaired T-test (two-tailed) was performed between male and female for each status and between status for males and females

**Figure SF2 (linked to Figure 2)**. Distribution of male patients with comorbidities according to age and Testosterone levels. Mann-Whitney test (two-tailed) was applied for pairwise group comparisons.

**Figure SF3 (linked to Figure 3)**. Longitudinal analysis of serum levels of IL-6, C-reactive protein (CRP), ferritin and lactate dehydrogenase (LDH) in male patients. For severe survivor and severe deceased outcomes, the trajectories of longitudinal determinations were submitted to linear regression analysis, and the resulting slopes compared for significance by two-way ANOVA. ns denotes not significant.


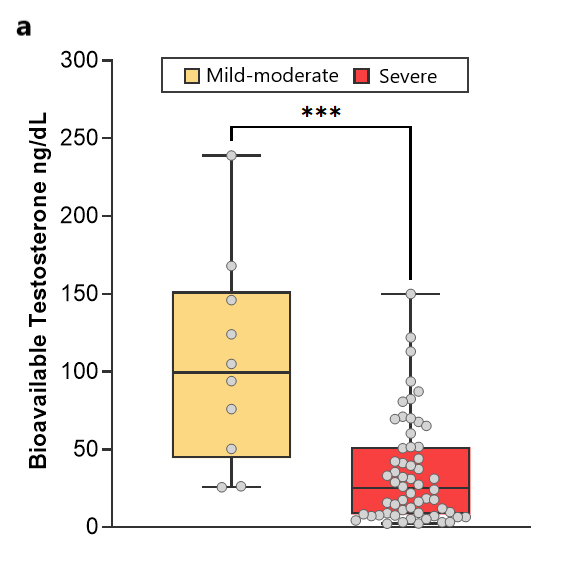


**
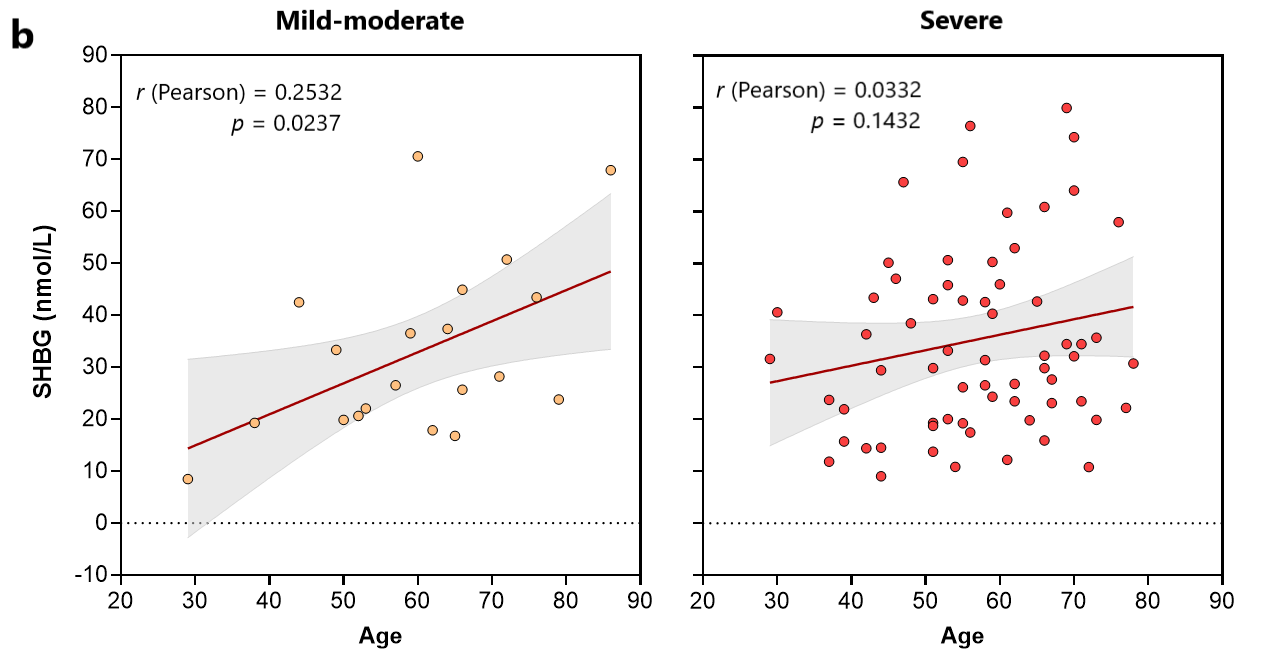
**

**Figure SF4.**  Bioavailable testosterone serum levels and correlation between age and sex-hormone binding globulin (SHBG). (**a)** Bioavailable testosterone levels were inferred from SHBG and albumin levels as described ^(27)^. There is a significant difference (*p* = 0.0002, Mann-Whitney test) between severe and mild-moderate outcomes. (**b**) Values for serum SHBG levels from 86 male patients were plotted against age. Significant correlation was observed for patients with mild-moderate (*r^2^* = 0.2532, *p* = 0.0237) but not severe outcomes.

**
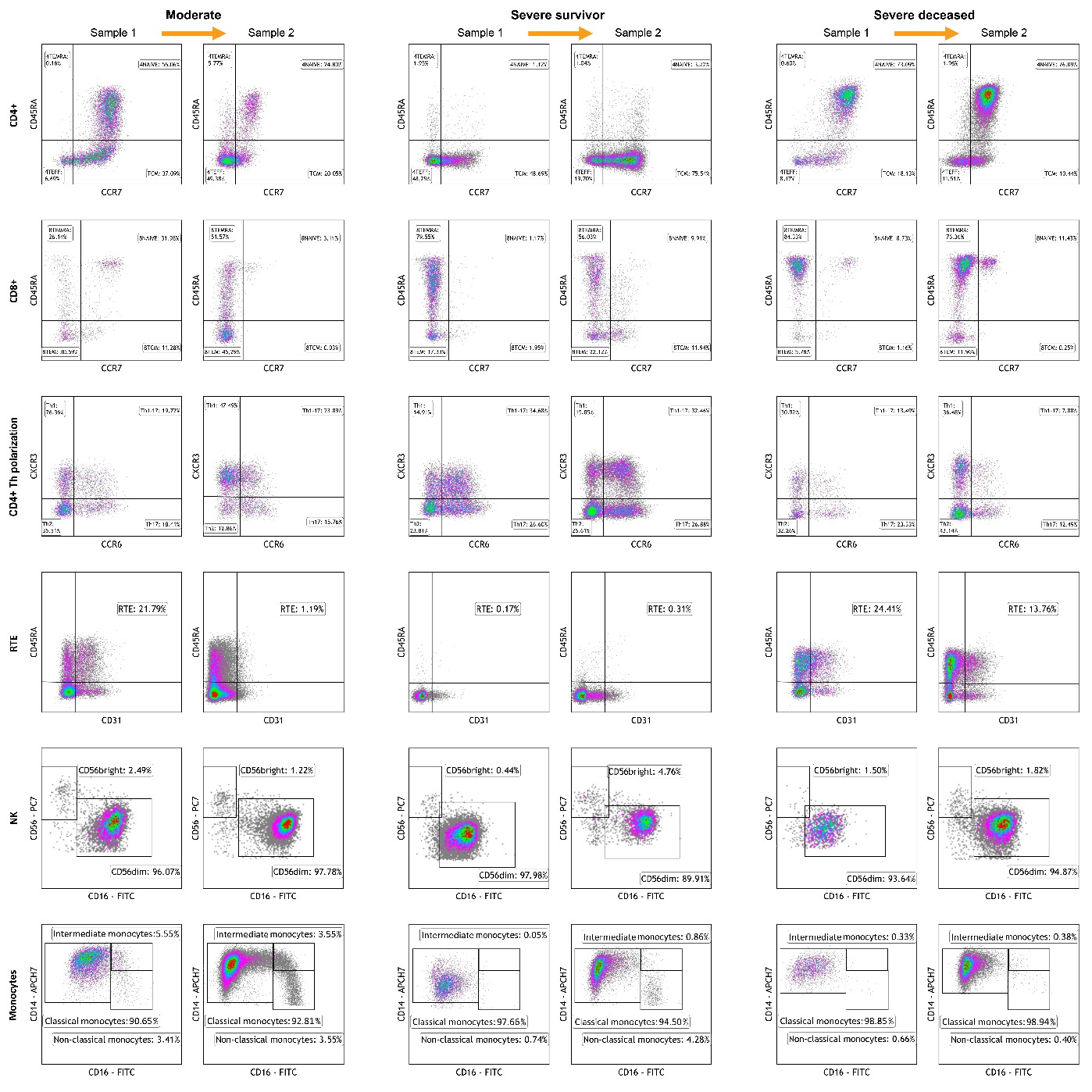
**

**Figure SF5 (linked to Figure 5)**. Flow cytometry analysis of circulating immune subpopulations in three illustrative cases with moderate, severe survivor and severe deceased outcomes. For each case, Sample 1 corresponds to the first available analysis post-admission, and Sample 2 corresponds to a subsequent analysis, separated from Sample 1 by 6-12 days. CD4+ and CD8+ subpopulations were further segmented into the indicated subpopulations by analyzing for CD45RA and CCR7 expression. CD3+ subpopulations were further segmented into subpopulations by analyzing the expression of the polarization markers CXCR3 and CCR6. Recent thymic emigrant (RTE) populations were identified and scored through the expression of CD3, CD45RA and CD31. Natural killer (NK) cell subpopulations were segmented by analyzing the expression of CD56 and CD16. Monocyte subpopulations were segmented by analyzing the expression of CD14 and CD16. Not shown are B cell and dendritic cell subpopulations, which did not show significant shifts in repertoire between outcomes and longitudinal sampling. 4TEFF, 4TCM, 4TEMRA, 8TEFF, 8TCM, 8TEMRA: CD4+ or CD8+ effector, central memory or T effector memory re-expressing CD45RA, respectively.
